# Synthesis and Antitumor Activities of Chiral Dipeptide Thioureas Containing an Alpha-Aminophosphonate Moiety

**DOI:** 10.3390/molecules22020238

**Published:** 2017-02-16

**Authors:** Jingzi Liu, Peng Liao, Junfeng Hu, Hong Zhu, Yonglin Wang, Yongjun Li, Yan Li, Bin He

**Affiliations:** 1 Engineering Research Center for the Development and Application of Ethnic Medicine and TCM (Ministry of Education), Guizhou Medical University, Guiyang 550004, Guizhou, China; m17785598945@163.com (P.L.); Zhhgmu@21cn.com (H.Z.); yonglinwang@gmc.edu.cn (Y.W.); m13765113985@163.com (Y.L.); 2 Guizhou Provincial Key Laboratory of Pharmaceutics, Guizhou Medical University, Guiyang 550004, Guizhou, China; 3 School of Pharmacy, Guizhou Medical University, Guiyang 550004, Guizhou, China; 4 National Engineering Research Center of Miao’s Medicines, Guizhou Medical University, Guiyang 550004, Guizhou, China; 5 School of Pharmacy, Zunyi Medical College, Zunyi 563000, Guizhou, China; hjfgmu@21cn.com; 6 School of Basic Medicine, Guizhou Medical University, Guiyang 550004, Guizhou, China

**Keywords:** dipeptide, thiourea, aminophosphonate, synthesis, antitumor agents

## Abstract

Thiourea derivatives demonstrate potent cytotoxic activity against various leukemias and many tumor cell lines. In our previous study, the combination of thiourea and phosphonate has been proven as an effective strategy for developing antitumor agents. Herein, we synthesized and evaluated a series of novel chiral dipeptide thioureas containing an α-aminophosphonate moiety as antitumor agents. Finally, we developed novel dipeptide thioureas **11d** and **11f** that showed comparable inhibition with that of Cisplatin against BGC-823 and A-549 cells, respectively.

## 1. Introduction

It is well-known that thiourea derivatives demonstrate potent cytotoxic activity against various leukemias and many tumor cell lines [1,2], and it is believed that this is due to their good inhibitory activity against protein tyrosine kinases (PTKs) [3,4,5,6], receptor tyrosine kinases (RTKs) [7], DNA topoisomerase [8,9,10], sirtuins [11], carbonic anhydrase [12], vanilloid receptor [13], a family of G protein-coupled receptors (sst1-5) [14], etc. In the past few decades, many thiourea derivatives have been reported on regarding their synthesis, evaluation as anticancer agents, structure-activity relationships, and mechanism of action [15,16,17,18]. For example, the derivatives show enhanced anticancer activities when thioureas are conjugated with amino acids [2]. We also combined amino acid and α-aminophosphonate to develop novel thiourea derivatives, called pseudo-peptide thioureas [19], which turned out to be another type of potential anticancer agents. Other functionalized thioureas were structurally incorporated with other scaffolds [2], such as benzensulfonamide [20], chlorocolchicine [21], podophyllotoxin [22], pyrazole [23], benzimidazole [24], benzothiazole [25], etc.

On the other hand, peptide conjugated derivatives have attracted much attention in recent years because of their broad biological activity, biocompatibility, and the possibility of introducing structural diversity in them, which became an effective strategy to obtain novel chemical entities when small bioactive motifs are conjugated with peptides [26]. Over the years, the conjugated molecules, as novel chemotherapeutics, have made significant progress due to the accessibility of combining the structural features of two or more small bioactive motifs to achieve novel molecules with enhanced bioactivities.

Based on our previous study [19], we would like to further introduce one more amino acid to the pseudo-peptide thiourea containing an α-aminophosphonate moiety. Herein, we describe the synthesis of a series of novel chiral dipeptide thioureas containing α-aminophosphonate moieties. The structures of the synthesized compounds were characterized by IR (Infrared spectrum), ^1^H-NMR (Nuclear Magnetic Resonance), ^13^C-NMR, ^31^P-NMR, ^19^F-NMR, and elemental analysis studies. The anticancer potency of all of these novel thioureas was examined in the human gastric cancer cell line BGC-823 and the human non-small cell lung cancer cell line A-549. To the best of our knowledge, this is the first report on the synthesis and antitumor activity of these dipeptide thioureas containing α-aminophosphonate moieties. These novel thioureas may provide promising lead compounds for treating human cancer. 

## 2. Results

**Figure 1:**
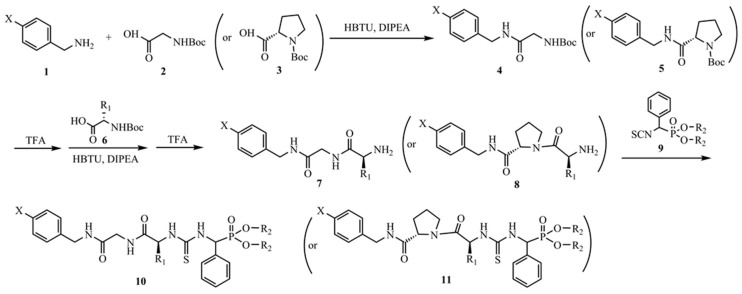
Synthetic route for novel thioureas **10** and **11**.

According to our pseudo-peptide thioureas containing α-aminophosphonate moieties described previously [19], we planned to introduce glycine or *L*-proline to obtain the novel thioureas **10** and **11** (Figure 1), respectively. The synthesis is outlined in Figure 1. Substituted benzylamine **1** was coupled with commercially available *N*-Boc protected glycine **2** or *N*-Boc protected *L*-proline **3** in the presence of *O*-benzotriazol-1-yl-*N*,*N*,*N’*,*N’*-tetramethyluronium hexafluorophosphate (HBTU). The Boc groups of the resulting compounds **4** and **5** were removed by trifluoroacetic acid (TFA) in CH_2_Cl_2_. Similarly, the dipeptides **7** and **8** were achieved by coupling with amino acid **6** after the deprotection. The key intermediate, *O*,*O’*-dialkylisothiocyanato (phenyl) methylphosphonate **9**, was prepared as described previously [19]. Finally, these novel thioureas **10** and **11** were obtained by nucleophilic addition of α-phosphonate isothiocyanate to the intermediate **9**. The structures, yields, and melting points of all these novel thioureas **10** and **11** are displayed in Table 1. All these novel thioureas were obtained in modest to excellent yields (from 53.9% to 98.4%).

With these novel thioureas in hands, we then selected two human cancer cell lines, BGC-823 and A-549, which are derived from stomach cancer and non-small cell lung cancer (NSCLC), respectively, and they were used to evaluate the anti-proliferation activities of thioureas **10** and **11** with a series concentration of 500 to 3.9 µM by comparison with a commercial anticancer drug, Cisplatin. As shown in Table 2, by the conventional 3-(4,5-dimethylthiazol-2-yl)-2,5-diphenyltetrazolium bromide (MTT) assay, thioureas **10** and **11** both demonstrated the capability of inhibiting the proliferation of BGC-823 and A549 cells with IC_50_ values in the range of 20.9 to 103.6 µM and 19.2 to 112.5 µM, respectively. Overall, thiourea **11** with the incorporation of *L*-proline demonstrated better anti-cancer activities in these two cancer cell lines than that of thiourea **10** with the incorporation of glycine (**10g**–**l** vs. **11a**–**f**, Table 2). Among the *O,O′*-Dialkylphosphonates derived from the ethyl, *n*-propyl, or *iso*-propyl groups of these compounds, different substituted phosphonate esters demonstrated no significant effect on the antitumor activities (Table 2). However, in some cases where R_2_ = *n*-propyl, the IC_50_ values were higher with respect to the other cases (for example: **10b** vs. **10a**,**c**; **10g** vs. **10h**,**i** for BGC-823, Table 2), which suggested that the n-propyl group may have a negative effect. Phenylalanine containing thiourea **10** or **11** (R_1_ = *L*-Ph) demonstrated better antitumor activity than that of phenylglycine containing thiourea **10** or **11** (R_1_ = *L*-Bn) (**10g**–**l** vs. **10a**–**f**, Table 2). Additionally, the *para*-fluorinated benzyl thioureas elicited superior antitumor activity to non-substituted benzyl thioureas (**11d**–**f** vs. **11a**–**c**; **10d**–**f** vs. **10a**–**c**; **10j**–**l** vs. **10g**–**I**; except for **11d** vs. **11a** for A549, Table 2). Notably, thiourea **11d** showed comparable inhibition with that of Cisplatin against BGC-823 cells (IC_50_ = 20.9 µM vs. 15.1 µM), while thiourea **11f** showed the highest inhibitory activity, close to that of Cisplatin, against A-549 cells (IC_50_ = 19.2 µM vs. 17.6 µM).

## 3. Materials and Methods

### 3.1. Materials

Reagents were obtained from Aldrich or Acros (Waltham, MA, USA) in the highest purity available and were used as supplied. The melting points of the products were determined on a XT-4 binocular microscope (Beijing Tech Instrument Co., Beijing, China) and were not corrected. The IR spectra were recorded on a Bruker VECTOR22 spectrometer in KBr disks (Billerica, MA, USA). ^1^H- and ^13^C-NMR spectra were recorded on a JEOL-300 NMR spectrometer (Otemachi, Chiyoda, Tokyo, Japan) at room temperature using tetramethylsilane (TMS) as an internal standard for ^1^H- and ^13^C-NMR while using H_3_PO_4_ (85%) and CFCl_3_ as an external reference for ^31^P- and ^19^F-NMR, respectively. The reported ^13^C chemical shifts are those present in the spectrum, and the ^31^P-^13^C coupling (or ^19^F-^13^C coupling) has not been considered. Elemental analysis was performed on an Elementar Vario-III CHN analyzer (Elementar, Frankfurt, Germany). UV spectra were recorded on a VARIAN Cary-50 spectrometer (Santa Clara, CA, USA) using a cell path length of 1 cm. BIO-RAD, Model 680 Microplate Reader (Hercules, CA, USA) was used to record those absorptions after MTT assay. The reagents were all of analytical grade or were chemically pure. Analytical thin layer chromatography (TLC) was performed on silica gel GF254.

### 3.2. Synthesis

A solution of *O*,*O*′-dialkylisothiocyanato (phenyl) methylphosphonate **9** [19] (1 mmol) in tetrahydrofuran (10 mL) was stirred, followed by drop wise addition of the intermediate **7** or **8** (1.1 mmol). The reaction mixture was stirred for 1 h at 23 °C, the solvent was removed by evaporation, and the crude product was purified by flash chromatography on silica using a mixture of petroleum ether and ethyl acetate as the eluent to yield the compounds **10a**–**l** and **11a**–**f** in 53.9%–98.4% yields. All final compounds were confirmed by ^1^H-NMR, ^13^C-NMR, IR, and elemental analysis. 

**10a:** C_29_H_35_N_4_O_5_PS, white solid, yield 91.6%, m.p. 179–180 °C, [α]${}_{D}^{20}$ = +57 (*c* = 0.12, CHCl_3_) IR ν: 3291, 3084, 3065, 3032, 2982, 2928, 2908, 1652, 1535, 1497, 1453, 1352, 1336, 1226, 1050, 1026 cm^−1^; ^1^H-NMR (300 MHz, CDCl_3_) δ: 8.61 (s, 1H, NH), 8.02 (s, 1H, NH), 7.93 (s, 1H, NH), 7.45–7.09 (m, 15H, ArH), 6.51 (dd, *J* = 22.3, 9.2 Hz, 1H, NH), 6.38 (dd, *J* = 22.2, 9.8 Hz, 1H, NCH-P), 4.93 (s, 1H, NCH), 4.18 (dd, *J* = 26.5, 4.8Hz, 4H, 2OCH_2_), 4.03–3.83 (m, 2H, NCH_2_-Ar), 3.70–3.52 (m, 2H, NCH_2_), 1.09 (t, *J* = 7.0 Hz, 3H, CH_3_), 1.03–0.92 (m, 3H, CH_3_) ; ^13^C-NMR (75 MHz, CDCl_3_) δ: 183.7, 171.5, 168.5, 137.9, 137.4, 135.1, 128.9, 128.5, 128.4, 127.6, 127.3, 63.4, 62.6, 61.9, 54.0, 43.4, 43.1, 16.1; ^31^P-NMR δ: 21.2 ppm; Anal. Calcd. (Analysis Calculated) for C_29_H_35_N_4_O_5_PS: C 60.17, H 5.72, N 9.99; Found: C 59.78, H 6.05, N 9.62.

**10b:** C_31_H_39_N_4_O_5_PS, white solid, yield 81.5%, m.p. 155–157 °C, [α]${}_{D}^{20}$ = +44 (*c* = 0.10, CHCl_3_) IR ν: 3295, 3083, 3064, 3031, 2969, 2935, 1654, 1541, 1497, 1454, 1355, 1227, 1204, 1058, 1012 cm^−1^; ^1^H-NMR (300 MHz, CDCl_3_) δ: 8.10 (d, *J* = 96.3 Hz, 2H, 2NH), 7.48–6.94 (m, 15H, ArH), 6.33 (s, 1H, NCH-P), 6.21 (s, 1H, NCH), 4.06 (dt, *J* = 82.5, 52.6 Hz, 2H, NCH_2_-Ar), 3.52 (s, 2H, NCH_2_), 3.31 (s, 2H, OCH_2_), 2.93 (s, 2H, OCH_2_), 1.57 (s, 4H, 2CH_2_), 1.40–1.35 (m, 2H, 2NH), 0.87 (d, *J* = 7.4 Hz, 6H, 2CH_3_); ^13^C-NMR (75 MHz, CDCl_3_) δ: 183.5, 171.5, 169.0, 138.0, 135.2, 129.0, 127.5, 127.1, 69.4, 69.0, 62.6, 53.8, 43.2, 42.1, 23.8, 23.6, 10.1, 9.9; ^31^P-NMR δ: 21.3 ppm; Anal. Calcd. for C_31_H_39_N_4_O_5_PS: C 60.60, H 6.61, N 8.79; Found: C 60.97, H 6.44, N 9.17.

**10c:** C_31_H_39_N_4_O_5_PS, white solid, yield 78.6%, m.p. 203–204 °C, [α]${}_{D}^{20}$ = +69 (*c* = 0.14, CHCl_3_) IR ν: 3323, 3269, 3089, 3031, 2980, 2932, 1678, 1639, 1537, 1512, 1453, 1224, 1002 cm^−1^; ^1^H-NMR (300 MHz, CDCl_3_) δ: 8.26 (s, 2H, 2NH), 8.03 (s, 2H, 2NH), 7.58–7.11 (m, 15H), 6.32 (d, *J* = 9.0 Hz, 1H, NCH-P), 4.73 (s, 1H, NCH), 4.49 (s, 2H, 2OCH), 4.31 (t, *J* = 30.3Hz, 2H, NCH_2_-Ar), 4.11 (d, *J* = 5.7 Hz, 2H, NCH_2_), 1.21–1.00 (m, 12H, 4CH_3_); ^13^C-NMR (75 MHz, CDCl_3_) δ: 171.4, 168.5, 138.0, 135.5, 129.0, 127.8, 127.3, 127.0, 72.7, 72.4, 61.8, 53.7, 43.4, 43.1, 24.4, 23.9, 23.6, 23.2; ^31^P-NMR δ: 19.4 ppm; Anal. Calcd. for C_31_H_39_N_4_O_5_PS: C 61.31, H 6.18, N 9.43; Found: C 60.97, H 6.44, N 9.17.

**10d:** C_29_H_34_FN_4_O_5_PS, white solid, yield 93.3%, m.p. 74–75 °C, [α]${}_{D}^{20}$ = +98 (*c* = 0.11, CHCl_3_) IR ν: 3294, 3065, 3033, 2987, 2931, 2910, 1673, 1539, 1510, 1221, 1204, 1049, 1024, 977 cm^−1^; ^1^H-NMR (300 MHz, CDCl_3_) δ: 8.68 (s, 1H, NH), 8.45 (s, 1H, NH), 7.48–7.06 (m, 12H, ArH), 7.00 (s, 1H, ArH), 6.88 (s, 1H, ArH), 6.79 (s, 1H, NCH-P), 5.26 (s, 1H, NCH), 4.33 (m, 2H, NCH_2_-Ar), 3.78 (m, 2H, NCH_2_), 3.59 (d, *J* = 6.8 Hz, 2H, OCH_2_), 3.39 (s, 2H, OCH_2_), 2.35 (s, 1H, NH), 2.01 (s, 1H, NH), 1.22 (t, *J* = 6.1 Hz, 3H, CH_3_), 0.98–0.85 (m, 3H, CH_3_); ^13^C-NMR (75 MHz, CDCl_3_) δ: 183.7, 171.5, 169.0, 160.8, 137.1, 135.0, 133.8, 129.6, 129.1, 128.45, 128.0, 127.5, 127.3, 115.4, 115.1, 63.5, 62.8, 62.2, 53.8, 42.7, 42.2, 14.3, 12.0; ^31^P-NMR δ: 21.1; ^19^F-NMR δ: −115.7 ppm; Anal. Calcd. for C_29_H_34_FN_4_O_5_PS: C 58.24, H 5.45, N 9.51; Found: C 57.99, H 5.71, N 9.33.

**10e:** C_31_H_38_FN_4_O_5_PS, white solid, yield 75.5%, m.p. 133–134 °C, [α]${}_{D}^{20}$ = +113 (*c* = 0.13, CHCl_3_) IR ν: 3290, 3066, 3036, 2972, 2938, 1673, 1540, 1511, 1454, 1352, 1337, 1223, 1062, 1010 cm^−1^; ^1^H-NMR (300 MHz, CDCl_3_) δ: 8.00 (s, 1H, NH), 7.82 (s, 1H, NH), 7.45–7.10 (m, 13H, ArH), 7.05 (s, 1H, ArH), 6.87 (d, *J* = 29.2 Hz, 2H, 2NH), 6.35 (s, 2H, NCH-P+ NCH), 4.42–3.95 (m, 2H, NCH_2_-Ar), 3.80 (s, 2H, NCH_2_), 3.64 (s, 2H, OCH_2_), 3.43 (d, *J* = 73.9 Hz, 2H, OCH_2_), 1.62 (s, 2H, CH_2_), 1.43 (d, *J* = 12.9 Hz, 2H, CH_2_), 0.79 (dt, *J* = 16.7, 7.4 Hz, 6H, 2CH_3_); ^13^C-NMR (75 MHz, CDCl_3_) δ: 183.8, 171.5, 168.6, 163.2, 137.1, 135.1, 129.5, 128.9, 128.7, 128.2, 128.1, 127.3, 115.4, 115.1, 69.5, 69.2, 62.7, 53.8, 42.1, 23.9, 23.6, 10.2, 10.0; ^31^P-NMR δ: 21.4; ^19^F-NMR δ: −115.8 ppm; Anal. Calcd. for C_31_H_38_FN_4_O_5_PS: C 59.36, H 5.87, N 9.12; Found: C 59.22, H 6.09, N 8.91.

**10f:** C_31_H_38_FN_4_O_5_PS, white solid, yield 77.8%, m.p. 176–177 °C, [α]${}_{D}^{20}$ = +85 (*c* = 0.14, CHCl_3_) IR ν: 3321, 3289, 3089, 3067, 2981, 2932, 1641, 1533, 1511, 1222, 999 cm^−1^; ^1^H-NMR (300 MHz, CDCl_3_) δ: 8.98 (s, 1H, NH), 8.45 (s, 1H, NH), 8.32 (d, *J* = 33.4 Hz, 2H, NH), 7.48–7.06 (m, 14H, ArH), 6.79 (s, 1H, NCH-P), 5.26 (s, 1H, NCH), 4.33 (m, 2H, NCH_2_Ar), 3.78 (m, 2H, NCH_2_), 3.59 (d, *J* = 6.8 Hz, 2H, OCH), 3.39 (s, 2H, OCH), 1.50–0.98 (m, 12H, 4CH_3_); ^13^C-NMR (75 MHz, CDCl_3_) δ: 183.6, 171.6, 168.6, 160.7, 138.2, 135.3, 133.9, 130.3, 129.5, 128.7, 127.9, 126.2, 115.9, 114.5, 73.4, 62.3, 55.3, 55.1, 43.1, 42.7, 24.7, 24.2, 23.6, 23.2; ^31^P-NMR δ: 19.5; ^19^F-NMR δ: −115.6 ppm; Anal. Calcd. for C_31_H_38_FN_4_O_5_PS: C 59.36, H 6.30, N 9.12; Found: C 59.22, H 6.09, N 8.91.

**10g:** C_30_H_37_N_4_O_5_PS, white solid, yield 66.8%, m.p. 167–168 °C, [α]${}_{D}^{20}$ = +47 (*c* = 0.10, CHCl_3_) IR ν: 3292, 3084, 3064, 3029, 2983, 2927, 1653, 1539, 1454, 1225, 1049, 1024, 976 cm^−1^; ^1^H-NMR (300 MHz, CDCl_3_) δ: 8.35 (s, 2H, 2NH), 8.04 (s, 2H, 2NH), 7.52–7.09 (m, 15H, ArH), 6.51 (s, 1H, NCH-P), 5.39 (d, *J* = 35.5 Hz, 1H, NCH), 4.39 (s, 2H, NCH-Ar), 4.10 (dd, *J* = 38.8, 23.9 Hz, 4H, 2OCH_2_), 3.77 (s, 1H, NCH), 3.57 (s, 1H, NCH), 2.99 (s, 1H, CHAr), 2.92 (s, 1H, CHAr), 1.06 (t, *J* = 7.0 Hz, 6H, 2CH_3_); ^13^C-NMR (75 MHz, CDCl_3_) δ: 183.2, 172.1, 168.2, 138.1, 136.5, 135.5, 129.4, 128.6, 128.1, 127.8, 127.3, 127.0, 63.7, 63.4, 59.8, 53.6, 43.4, 36.6, 16.2; ^31^P-NMR δ: 21.3 ppm; Anal. Calcd. for C_30_H_37_N_4_O_5_PS: C 60.61, H 6.07, N 9.86; Found: C 60.39, H 6.25, N 9.39.

**10h:** C_32_H_41_N_4_O_5_PS, white solid, yield 71.4%, m.p. 148–149 °C, [α]${}_{D}^{20}$ = +54 (*c* = 0.12, CHCl_3_) IR ν: 3294, 3084, 3064, 3031, 2969, 2935, 1654, 1542, 1497, 1454, 1355, 1336, 1227, 1056, 1007 cm^−1^; ^1^H-NMR (300 MHz, CDCl_3_) δ: 8.32 (d, *J* = 44.4 Hz, 2H, 2NH), 7.99 (s, 1H, NH), 7.51–7.03 (m, 15H, ArH), 6.92 (s, 1H, NH), 6.49 (s, 1H, NCH-P), 5.29 (s, 1H, NCH), 4.47 (m, 2H, NCH_2_-Ar), 4.38 (m, 2H, NCH_2_), 3.82 (m, 2H, OCH_2_), 3.62 (d, *J* = 30.8 Hz, 2H, OCH_2_), 3.19–2.85 (m, 2H, CH_2_Ar), 1.61 (d, *J* = 11.1 Hz, 2H, CH_2_), 1.49–1.29 (m, 2H, CH_2_), 0.98–0.79 (m, 3H, CH_3_), 0.71 (t, *J* = 7.3 Hz, 3H, CH_3_); ^13^C-NMR (75 MHz, CDCl_3_) δ: 183.2, 172.0, 168.1, 138.1, 136.5, 135.6, 129.4, 128.7, 128.2, 127.8, 127.5, 127.1, 69.2, 68.7, 53.8, 43.4, 39.5, 23.7, 10.3, 10.1; ^31^P-NMR δ: 21.4 ppm; Anal. Calcd. for C_32_H_41_N_4_O_5_PS: C 61.15, H 6.87, N 9.04; Found: C 61.52, H 6.61, N 8.97.

**10i:** C_32_H_41_N_4_O_5_PS, white solid, yield 70.4%, m.p. 72–73 °C, [α]${}_{D}^{20}$ = +76 (*c* = 0.11, CHCl_3_) IR ν: 3296, 3085, 3064, 3031, 2981, 2932, 1651, 1536, 1225, 998 cm^−1^; ^1^H-NMR (300 MHz, CDCl_3_) δ: 8.35 (s, 2H, 2NH), 8.04 (s, 2H, 2NH), 7.38–6.90 (m, 15H, ArH), 6.51 (s, 1H, NCH-P), 5.39 (d, *J* = 35.5 Hz, 1H, NCH), 4.39 (s, 2H, NCH-Ar), 3.99 (d, *J* = 4.4 Hz, 2H, 2OCH), 3.77 (s, 1H, NCH), 3.57 (s, 1H, NCH), 2.99 (s, 1H, CHAr), 2.92 (s, 1H, CHAr), 1.06 (t, *J* = 7.0Hz, 6H, 2CH_3_) ; ^13^C-NMR (75 MHz, CDCl_3_) δ: 183.1, 171.8, 171.2, 137.9, 136.3, 135.7, 129.2, 128.6, 128.3, 128.1, 127.5, 127.1, 126.9, 72.7, 72.3, 60.4, 53.6, 43.3, 42.6, 39.2, 24.1, 23.7, 23.0; ^31^P-NMR δ: 19.6 ppm; Anal. Calcd. for C_32_H_41_N_4_O_5_PS: C 61.91, H 6.28, N 9.35; Found: C 61.52, H 6.61, N 8.97.

**10j:** C_30_H_36_FN_4_O_5_PS, white solid, yield 98.7%, m.p. 75–76 °C, [α]${}_{D}^{20}$ = +107 (*c* = 0.12, CHCl_3_) IR ν: 3291, 3077, 3065, 3032, 2987, 2931, 1673, 1615, 1542, 1511, 1221, 1049, 1025, 976 cm^−1^; ^1^H-NMR (300 MHz, CDCl_3_) δ: 8.24 (s, 1H, NH), 7.97 (s, 1H, NH), 7.47–6.77 (m, 14H), 6.44 (s, 1H, NCH-P), 5.28 (s, 1H, NCH), 4.36–3.84 (m, 4H, NCH_2_Ar+ NCH_2_), 3.60 (d, *J* = 56.9 Hz, 4H, 2OCH_2_), 3.03 (d, *J* = 37.2 Hz, 2H, CH_2_-Ar), 2.19–1.86 (m, 2H, 2NH), 1.24 (dd, *J* = 13.2, 6.2 Hz, 3H, CH_3_), 1.01 (d, *J* = 1. 8 Hz, 3H, CH_3_) ; ^13^C-NMR (75 MHz, CDCl_3_) δ: 183.4, 171.9, 171.4, 163.3, 136.4, 135.5, 134.0, 129.8, 128.9, 128.5, 128.1, 127.1, 115.4, 115.2, 60.6, 60.1, 53.7, 42.8, 42.1, 39.1, 17.6, 14.4; ^31^P-NMR δ: 21.1; ^19^F-NMR δ: −115.5 ppm; Anal. Calcd. for C_30_H_36_FN_4_O_5_ PS: C 58.35, H 6.13, N 8.87; Found: C 58.62, H 5.90, N 9.11.

**10k:** C_32_H_40_FN_4_O_5_PS, white solid, yield 94.3%, m.p. 95–96 °C, [α]${}_{D}^{20}$ = +131 (*c* = 0.13, CHCl_3_) IR ν: 3294, 3066, 3032, 2970, 2935, 1653, 1540, 1511, 1454, 1353, 1338, 1223, 1062, 1009 cm^−1^; ^1^H-NMR (300 MHz, CDCl_3_) δ: 8.27 (s, 2H, 2NH), 7.82 (s, 2H, 2NH), 7.56–6.77 (m, 14H, ArH), 6.49 (s, 1H, NCH-P), 5.30 (s, 1H, NCH), 4.39 (s, 2H, NCH_2_-Ar), 3.89–3.78 (m, 4H, 2OCH_2_), 3.51 (d, *J* = 7.2 Hz, 2H, NCH_2_), 3.02 (d, *J* = 54.6 Hz, 2H, CH_2_-Ar), 1.62 (d, *J* = 10.4 Hz, 2H, CH_2_), 1.50–1.41 (m, 2H, CH_2_), 1.00–0.83 (m, 3H, CH_3_), 0.73 (t, *J* = 6.2 Hz, 2H, CH_3_); ^13^C-NMR (75 MHz, CDCl_3_) δ: 183.3, 171.9, 168.2, 160.9, 136.4, 135.6, 133.9, 129.7, 129.3, 128.8, 128.2, 127.2, 115.5, 115.2, 69.3, 69.2, 60.1, 53.8, 42.7, 39.4, 24.0, 23.8, 10.1; ^31^P-NMR δ: 21.4; ^19^F-NMR δ: −115.6; ppm; Anal. Calcd. for C_32_H_40_FN_4_O_5_PS: C 59.59, H 6.01, N 8.94; Found: C 59.80, H 6.27, N 8.72.

**10l:** C_32_H_40_FN_4_O_5_PS, white solid, yield 95.0%, m.p. 62–63 °C, [α]${}_{D}^{20}$ = +86 (*c* = 0.12, CHCl_3_) IR ν: 3300, 3066, 3032, 2981, 2931, 2878, 1651, 1540, 1511, 1224, 1102, 999 cm^−1^; ^1^H-NMR (300 MHz, CDCl_3_) δ: 8.37 (s, 1H, NH), 8.12 (s, 1H, NH), 7.48–6.97 (m, 12H), 6.86 (d, *J* = 20.7 Hz, 2H, 2ArH), 4.53 (s, 1H, NCH-P), 4.28 (s, 1H, NCH), 4.07 (dd, *J* = 14.3, 7.2 Hz, 2H, NCH_2_-Ar + NCH_2_), 3.97 (s, 2H, 2OCH), 2.97 (d, *J* = 56.4 Hz, 2H, CH_2_Ar), 2.29 (s, 2H, 2NH), 1.27–1.11 (m, 12H, 4CH_3_); ^13^C-NMR (75 MHz, CDCl_3_) δ: 183.3, 171.9, 171.3, 163.2, 136.4, 135.9, 134.0, 129.4, 128.5, 128.1, 127.0, 115.4, 115.2, 72.7, 60.5, 53.6, 42.7, 39.3, 21.2; ^31^P-NMR δ: 19.3; ^19^F-NMR δ: −115.4 ppm; Anal. Calcd. for C_32_H_40_FN_4_O_5_PS: C 59.62, H 6.46, N 8.47; Found: C 59.80, H 6.27, N 8.72.

**11a:** C_33_H_41_N_4_O_5_PS, white solid, yield 92.6%, m.p. 103–104 °C, [α]${}_{D}^{20}$ = +115 (*c* = 0.12, CHCl_3_) IR ν: 3300, 3087, 3064, 3031, 2985, 2928, 2888, 1677, 1542, 1453, 1394, 1357, 1203, 1025 cm^−1^; ^1^H-NMR (300 MHz, CDCl_3_) δ: 8.81 (s, 1H), 8.58 (s, 1H), 8.43 (s, 1H), 7.47–6.98 (m, 15H), 6.27 (s, 1H), 5.40 (s, 1H), 4.54 (d, *J* = 57.8 Hz, 2H), 4.24 (s, 1H), 3.78 (d, *J* = 6.5 Hz, 2H), 3.60 (dd, *J* = 10.1, 6.6 Hz, 4H), 3.02 (d, *J* = 3.3 Hz, 2H), 1.92 (dd, *J* = 68.6, 32.5 Hz, 4H), 0.99 (dt, *J* = 28.1, 6.9 Hz, 6H); ^13^C-NMR (75 MHz, CDCl_3_) δ: 183.3, 172.2, 171.5, 138.6, 136.1, 135.9, 129.4, 128.83, 128.2, 128.0, 127.7, 127.5, 126.8, 64.0, 63.5, 60.2, 57.6, 53.6, 43.0, 42.0, 38.6, 24.9, 18.4, 11.9; ^31^P-NMR δ: 21.5 ppm; Anal. Calcd. for C_33_H_41_N_4_O_5_PS: C 62.41, H 6.85, N 8.46; Found: C 62.25, H 6.49, N 8.80.

**11b:** C_35_H_45_N_4_O_5_PS, white solid, yield 90.2%, m.p. 117–118 °C, [α]${}_{D}^{20}$ = +133 (*c* = 0.13, CHCl_3_) IR ν: 3423, 3087, 3060, 3033, 2993, 2955, 2887, 1676, 1542, 1456, 1405, 1364, 1204, 1030 cm^−1^; ^1^H-NMR (300 MHz, CDCl_3_) δ: 8.47 (d, *J* = 5.5 Hz, 2H), 8.23 (s, 1H), 7.53 (s, 1H), 7.33 (s, 1H), 7.27–6.99 (m, 13H), 6.35 (s, 1H), 5.23 (s, 1H), 4.33 (d, *J* = 19.6 Hz, 2H), 4.20 (s, 1H), 3.62–3.50 (m, 4H), 3.35 (d, *J* = 26.1 Hz, 2H), 3.10 (s, 2H), 2.75–2.56 (m, 4H), 1.80 (dd, *J* = 71.0, 33.4 Hz, 3H), 1.49–1.33 (m, 3H); ^13^C-NMR (75 MHz, CDCl_3_) δ: 170.8, 161.2, 160.8, 138.5, 134.3, 129.6, 129.2, 128.9, 128.4, 128.2, 127.6, 127.2, 126.7, 118.2, 115.3, 60.5, 58.8, 56.0, 53.4, 52.9, 42.8, 41.9, 38.3, 28.1, 24.6, 22.1, 17.5, 11.7; ^31^P-NMR δ: 21.2 ppm; Anal. Calcd. for C_35_H_45_N_4_O_5_PS: C 63.10, H 6.66, N 8.62; Found: C 63.23, H 6.82, N 8.43.

**11c:** C_35_H_45_N_4_O_5_PS, white solid, yield 81.8%, m.p. 89–90 °C, [α]${}_{D}^{20}$ = +89 (*c* = 0.12, CHCl_3_) IR ν: 3301, 3085, 3064, 3031, 2983, 2935, 2884, 1675, 1542, 1454, 1386, 1359, 1203, 999 cm^−1^; ^1^H-NMR (300 MHz, CDCl_3_) δ: 8.83 (d, *J* = 12.3 Hz, 2H), 8.30 (s, 1H), 7.98–6.59 (m, 15H), 6.19 (s, 1H), 5.53 (s, 1H), 4.68 (d, *J* = 13.0 Hz, 2H), 4.46 (s, 1H), 4.33 (d, *J* = 15.4 Hz, 2H), 4.16 (s, 1H), 3.36 (s, 1H), 3.23 (s, 1H), 3.05 (s, 1H), 1.98 (dd, *J* = 81.3, 17.0 Hz, 4H), 1.40–0.88 (m, 12H); ^13^C-NMR (75 MHz, CDCl_3_) δ: 183.6, 172.7, 172.6, 139.1, 139.1, 136.6, 129.8, 129.6, 128.8, 128.5, 128.4, 128.4, 127.3, 127.2, 126.7, 72.8, 72.4, 60.3, 57.8, 55.8, 48.2, 43.0, 39.1, 29.6, 25.1, 24.3, 23.5; ^31^P-NMR δ: 19.9 ppm; Anal. Calcd. for C_35_H_45_N_4_O_5_PS: C 63.48, H 6.66, N 8.71; Found: C 63.23, H 6.82, N 8.43.

**11d:** C_33_H_40_FN_4_O_5_PS, white solid, yield 93.5%, m.p. 97–98 °C, [α]${}_{D}^{20}$ = +121 (*c* = 0.11, CHCl_3_) IR ν: 3302, 3085, 3063, 3031, 2990, 2953, 2887, 1680, 1542, 1453, 1404, 1357, 1203, 1026, 720; ^1^H-NMR (300 MHz, CDCl_3_) δ: 9.03–8.50 (m, 2H), 8.33 (s, 1H), 7.19–6.92 (m, 12H), 6.72 (d, *J* = 8.7 Hz, 2H), 6.14 (s, 1H), 5.22 (s, 1H), 4.56–4.02 (m, 3H), 3.87 (d, *J* = 7.1 Hz, 2H), 3.64 (dd, *J* = 19.5, 10.5 Hz, 4H), 3.15 (s, 1H), 2.96 (d, *J* = 13.5 Hz, 1H), 1.81 (d, *J* = 9.7 Hz, 2H), 1.69 (d, *J* = 30.6 Hz, 1H), 0.91–0.81 (td, *J* = 16.0, 7.9 Hz, 6H); ^13^C-NMR (75 MHz, CDCl_3_) δ: 183.0, 171.6, 170.7, 160.5, 135.8, 135.4, 134.2, 128.5, 128.1, 127.6, 127.3, 126.5, 114.7, 114.5, 63.6, 63.1, 63.0, 59.9, 57.4, 41.8, 38.1, 29.1, 24.6, 20.6, 15.7, 13.8; ^31^P-NMR δ: 21.2; ^19^F-NMR δ: −115.8 ppm; Anal. Calcd. for C_33_H_40_FN_4_O_5_PS: C 60.40, H 6.37, N 8.95; Found: C 60.54, H 6.16, N 8.56.

**11e:** C_35_H_44_FN_4_O_5_PS, white solid, yield 53.9%, m.p. 107–108 °C, [α]${}_{D}^{20}$ = +137 (*c* = 0.13, CHCl_3_) IR ν: 3296, 3087, 3065, 3031, 2970, 2932, 2882, 1662, 1539, 1454, 1383, 1357, 1222, 1012; ^1^H-NMR (300 MHz, CDCl_3_) δ: 8.66–8.49 (m, 2H), 8.35 (s, 1H), 7.45–7.05 (m, 12H), 6.92 (d, *J* = 8.4 Hz, 2H), 6.10 (s, 1H), 5.25 (s, 1H), 4.51 (s, 1H), 4.30 (d, *J* = 46.8 Hz, 2H), 3.98 (d, *J* = 58.8 Hz, 2H), 3.53 (dd, *J* = 51.6, 32.8 Hz, 4H), 3.05 (s, 1H), 2.92 (s, 1H), 2.20 (s, 1H), 1.96–1.75 (m, 3H), 1.38 (dd, *J* = 17.7, 7.2 Hz, 2H), 1.21 (t, *J* = 7.1 Hz, 3H), 0.98–0.60 (m, 3H); ^13^C-NMR (75 MHz, CDCl_3_) δ: 173.1, 171.0, 169.5, 160.8, 136.4, 136.1, 134.3, 129.2, 128.6, 127.2, 115.5, 115.3, 69.2, 68.8, 60.3, 59.1, 54.3, 47.3, 45.4, 36.8, 28.4, 25.0, 22.5, 21.1, 10.0, 9.9; ^31^P-NMR δ: 21.6; ^19^F-NMR δ: −115.9 ppm; Anal. Calcd. for C_35_H_44_FN_4_O_5_PS: C 61.34, H 6.77, N 8.02; Found: C 61.57, H 6.50, N 8.21.

**11f:** C_35_H_44_FN_4_O_5_PS, white solid, yield 82.4%, m.p. 83–84 °C, [α]${}_{D}^{20}$ = +107 (*c* = 0.12, CHCl_3_) IR ν: 3299, 3087, 3066, 3031, 2980, 2934, 2879, 1676, 1542, 1453, 1386, 1353, 1223, 999; ^1^H-NMR (300 MHz, CDCl_3_) δ: 8.73–8.55 (s, 2H), 8.34 (s, 1H), 7.58–7.01 (m, 12H), 6.88 (d, *J* = 12.5 Hz, 2H), 6.17 (s, 1H), 5.45 (s, 1H), 4.54 (d, *J* = 37.1 Hz, 2H), 4.30 (s, 1H), 4.06 (d, *J* = 7.2 Hz, 2H), 3.62 (s, 1H), 3.46 (s, 1H), 3.03 (d, *J* = 7.5 Hz, 2H), 2.03–1.83 (m, 4H), 1.41–1.30 (m, 3H), 1.20 (t, *J* = 7.1 Hz, 3H), 1.17–1.08 (m, 3H), 0.86–0.81 (m, 3H); ^13^C-NMR (75 MHz, CDCl_3_) δ: 183.3, 172.3, 171.2, 160.6, 136.4, 136.0, 135.9, 128.9, 128.5, 127.7, 126.9, 115.2, 115.1, 72.6, 72.5, 60.4, 57.6, 53.6, 42.3, 42.0, 38.9, 28.3, 24.9, 24.2, 24.1, 23.2, 21.1; ^31^P-NMR δ: 19.8; ^19^F-NMR δ: −115.7 ppm; Anal. Calcd. for C_35_H_44_FN_4_O_5_PS: C 61.36, H 6.82, N 8.00; Found: C 61.57, H 6.50, N 8.21.

### 3.3. Antitumor Activity

All tested compounds were dissolved in DMSO and subsequently diluted in the culture medium in indicated final concentrations before treatment of the cultured cells. Tested cells were plated in 96-well plates at a density 2 × 10^4^ cells/well/100 µL of the proper culture medium and were treated with the compounds at 3.9–500 µM for 72 h. In parallel, the cells treated with 0.1% DMSO served as a control. An MTT [3-(4,5-dimethylthiazol-2-yl)-2,5-diphenyltetrazolium bromide] assay was performed 4 h later, according to the instructions. This assay was based on the cellular cleavage of MTT into formazane which is soluble in the cell culture medium. Any absorbance caused by formazan was measured at 595 nm with a microplate reader (BIO-RAD, model 680), which was directly proportional to the number of living cells in the culture. Two types of cells were used in these assays, BGC-823 (human gastric cancer) and A-549 (non-small cell lung cancer) cell lines, provided by ATCC (American Type Culture Collection) and cultivated in RPMI 1640 (for BGC-823 and A-549) supplemented with 10% fetal bovine serum. Tissue culture reagents were obtained from Gibco BRL. The experiment was performed in triplicate.

## 4. Conclusions

In summary, this study is based on our previous work [19]. Our attempt was to incorporate glycine or rigid amino acids such as *L*-proline into the previous pseudo-peptide thiourea containing an α-aminophosphonate moiety. A series of novel chiral dipeptide thioureas containing α-aminophosphonate moieties **10a**–**l** and **11a**–**f** were synthesized in high yield (Table 1). All these novel thioureas could inhibit tumor cell lines (BGC-823 and A-549) below 100 µM (Table 2) by the MTT assay. Although MTT is not a direct reflection on cell proliferation specifically and other orthogonal assays may need to be performed [27], the thioureas **11d** and **11f** showed comparable inhibition with that of Cisplatin against BGC-823 and A-549 cells, respectively (Table 2). Finally, we can conclude their structure-activity relationship; overall, the rigid amino acid of incorporated *L*-proline is helpful for antitumor activity. *L*-phenylalanine containing thioureas also showed better antitumor activities than that of *L*-phenylglycine containing thioureas. Our results also indicate that the antitumor activity could be improved by introducing an electron-withdrawing group in the *para* position of the terminal phenyl group of the dipeptide thioureas. This will guide us to design and obtain more and more potent antitumor agents. The mechanism of antitumor activity for these novel dipeptide thioureas is under further investigation.

## Figures and Tables

**Table 1:** Structure, yield, and melting point of novel thioureas **10** and **11**.

Compound	X	R_1_	R_2_	Yield (%)	m.p. (°C)
**10a**	H	*L*-Ph	Et	91.6	179–180
**10b**	H	*L*-Ph	*n*-Pr	81.5	155–157
**10c**	H	*L*-Ph	*i*-Pr	78.6	203–204
**10d**	4-F	*L*-Ph	Et	93.3	74–75
**10e**	4-F	*L*-Ph	*n*-Pr	75.5	133–134
**10f**	4-F	*L*-Ph	*i*-Pr	77.8	176–177
**10g**	H	*L*-Bn	Et	66.8	167–168
**10h**	H	*L*-Bn	*n*-Pr	71.4	148–149
**10i**	H	*L*-Bn	*i*-Pr	70.4	72–73
**10j**	4-F	*L*-Bn	Et	98.7	75–76
**10k**	4-F	*L*-Bn	*n*-Pr	94.3	95–96
**10l**	4-F	*L*-Bn	*i*-Pr	95.0	62–63
**11a**	H	*L*-Bn	Et	92.6	103–104
**11b**	H	*L*-Bn	*n*-Pr	90.2	117–118
**11c**	H	*L*-Bn	*i*-Pr	81.8	89–90
**11d**	4-F	*L*-Bn	Et	93.5	97–98
**11e**	4-F	*L*-Bn	*n*-Pr	53.9	107–108
**11f**	4-F	*L*-Bn	*i*-Pr	82.4	83–84

**Table 2:** IC_50_ values of thioureas **10** and **11** for BGC-823 and A-549 cells.

Compound	X	R_1_	R_2_	IC_50_/(μmol·L^−1^)	
				BGC-823	A-549
**10a**	H	*L*-Ph	Et	54.8 ± 3.2	63.2 ± 2.1
**10b**	H	*L*-Ph	*n*-Pr	87.3 ± 7.1	112.5 ± 7.9
**10c**	H	*L*-Ph	*i*-Pr	61.5 ± 2.2	74.3 ± 6.6
**10d**	4-F	*L*-Ph	Et	51.9 ± 3.5	51.5 ± 3.8
**10e**	4-F	*L*-Ph	*n*-Pr	58.1 ± 2.0	56.2 ± 4.2
**10f**	4-F	*L*-Ph	*i*-Pr	47.2 ± 4.3	43.4 ± 3.1
**10g**	H	*L*-Bn	Et	53.5 ± 1.8	67.5 ± 3.4
**10h**	H	*L*-Bn	*n*-Pr	103.6 ± 8.9	58.3 ± 4.1
**10i**	H	*L*-Bn	*i*-Pr	42.7 ± 2.1	46.1 ± 3.3
**10j**	4-F	*L*-Bn	Et	38.3 ± 3.3	35.2 ± 2.5
**10k**	4-F	*L*-Bn	*n*-Pr	44.1 ± 3.1	49.5 ± 5.1
**10l**	4-F	*L*-Bn	*i*-Pr	24.8 ± 2.6	41.7 ± 3.0
**11a**	H	*L*-Bn	Et	31.4 ± 2.0	23.7 ± 2.2
**11b**	H	*L*-Bn	*n*-Pr	45.2 ± 1.6	34.6 ± 2.1
**11c**	H	*L*-Bn	*i*-Pr	39.7 ± 3.3	40.8 ± 3.9
**11d**	4-F	*L*-Bn	Et	20.9 ± 2.8	30.3 ± 1.8
**11e**	4-F	*L*-Bn	*n*-Pr	37.5 ± 3.7	29.5 ± 3.4
**11f**	4-F	*L*-Bn	*i*-Pr	25.6 ± 4.1	19.2 ± 2.3
**Cisplatin**				15.1 ± 2.3	17.6 ± 3.1
